# Small-bowel B-cell lymphoma presenting as autoimmune hemolytic anemia and severe obscure gastrointestinal bleeding

**DOI:** 10.1055/a-2098-0883

**Published:** 2023-07-11

**Authors:** Katarzyna M. Pawlak, Alvaro Martínez-Alcalá, Paul Thomas Kröner, Lucía C. Fry, Klaus Mönkemüller

**Affiliations:** 1 Department of Gastroenterology, Hospital of the Ministry of Internal Affairs, Szczecin, Poland; 2 Department of Gastroenterology, Helios Frankenwaldklinik, Kronach, Germany; 3 Department of Gastroenterology, Ameos Teaching University Hospital (Otto-von-Guericke University – Magdeburg), Halberstadt, Germany; 4 University of Belgrade, Belgrade, Serbia; 5 Department of Gastroenterology “Prof. Carolina Olano”, Hospital de Clínicas, Universidad de la República, Montevideo, Uruguay

A 75-year-old man was admitted to our hospital because of worsening chronic hemolytic anemia and hematochezia. His medical history was significant for diabetes mellitus, coronary artery disease, and autoimmune hemolytic anemia (AIHA). Idiopathic AIHA was diagnosed 3 years prior to the current admission and was warm antibody-mediated, and direct antiglobulin test (Coombs test) positive. Initially he was placed on steroids, but due to recurrent hemolytic episodes, he was subsequently placed on different immunosuppressive agents, including azathioprine, cyclophosphamide, and mycophenolate mofetil.

Esophagogastroduodenoscopy and colonoscopy were unremarkable. Capsule endoscopy showed an ulcerated tumor located in the ileum ( Fig. 1 a ). A retrograde deep enteroscopy confirmed a large tumor in the ileum ( Fig. 1 b ). The patient underwent surgical resection of the mass ( Fig. 1 c ). Histopathology revealed diffuse large B-cell lymphoma (DLBCL), an aggressive subtype of non-Hodgkin lymphoma (NHL) ( Fig. 1 d–f , Video 1 ).

**Fig. 1:**
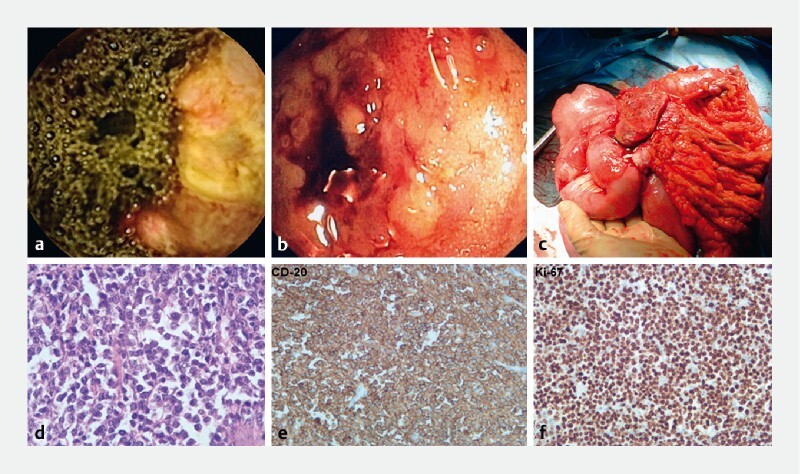
Patient investigations. **a** Capsule endoscopy showing an ulcerated mass. **b** On enteroscopy, a stenotic, ulcerated, and bleeding mass was evident. **c** Operative photograph showing a large mass involving the terminal ileum. **d** Hematoxylin and eosin stain revealed neoplastic lymphoid cells. **e** The CD-20 marker confirmed the B-cell origin of the lymphoma. **f** Positive staining for Ki-67 in more than 90 % of the cells.

**Video 1** Small-bowel B-cell lymphoma presenting as autoimmune hemolytic anemia and severe obscure gastrointestinal bleeding.


The Coombs test was negative 2 months later, suggesting that the mass was the source of the autoantibodies. Combination R-CHOP therapy (rituximab, cyclophosphamide, doxorubicin hydrochloride, vincristine sulfate, and prednisone) resulted in remission of the lymphoma.

This case highlights the occurrence of two conditions leading to severe anemia. Whenever an AIHA is present, the clinician must be aware of lymphomas. Interestingly, AIHA may occur prior to NHL (from 3 months to 13 years before lymphoma diagnosis), concurrent with (6 months before until 6 months after NHL diagnosis), at relapse of NHL, or in complete remission after successful treatment of the NHL; however, the majority of cases occur concurrently 1 2 3 . The most common primary small intestine lymphoma is DLBCL (58 % of cases) and perforation is a frequent complication. Endoscopically, AIHA can be of polypoid type in 25 % of cases, ulcerative type in 54 %, multiple polyposis in 5 %, diffuse-infiltrating type in 6 %, and mixed type in 10 % of patients 2 3 .

Endoscopy_UCTN_Code_CCL_1AC_2AC

## Notes

Endoscopy E-Videos

https://eref.thieme.de/e-videos
*E-Videos* is an open access online section of the journal *Endoscopy* , reporting on interesting cases and new techniques in gastroenterological endoscopy. All papers include a high-quality video and are published with a Creative Commons CC-BY license. Endoscopy E-Videos qualify for HINARI discounts and waivers and eligibility is automatically checked during the submission process. We grant 100% waivers to articles whose corresponding authors are based in Group A countries and 50% waivers to those who are based in Group B countries as classified by Research4Life (see: https://www.research4life.org/access/eligibility/ ). This section has its own submission website at https://mc.manuscriptcentral.com/e-videos
